# New tools for detecting latent tuberculosis infection: evaluation of RD1-specific long-term response

**DOI:** 10.1186/1471-2334-9-182

**Published:** 2009-11-21

**Authors:** Ornella Butera, Teresa Chiacchio, Stefania Carrara, Rita Casetti, Valentina Vanini, Serena Meraviglia, Giuliana Guggino, Francesco Dieli, Marco Vecchi, Francesco N Lauria, Almerico Marruchella, Patrizia Laurenti, Mahavir Singh, Nadia Caccamo, Enrico Girardi, Delia Goletti

**Affiliations:** 1 Translational Research Unit, Department of Epidemiology and Preclinical Research, L. Spallanzani National Institute for Infectious Diseases (INMI), IRCCS, Rome, Italy; 2 Cellular Immunology Laboratory (INMI), Italy; 3 Biopathology Department, Palermo University, Italy; 4 Health Department (INMI), Italy; 5 Hygiene Department, Catholic University of Rome, Italy; 6 LIONEX GmbH, Braunschweig, Germany; 7 Epidemiology Unit, Department of Epidemiology and Preclinical Research (INMI), Italy

## Abstract

**Background:**

Interferon-gamma (IFN-γ) release assays (IGRAs) were designed to detect latent tuberculosis infection (LTBI). However, discrepancies were found between the tuberculin skin test (TST) and IGRAs results that cannot be attributed to prior Bacille Calmètte Guerin vaccinations. The aim of this study was to evaluate tools for improving LTBI diagnosis by analyzing the IFN-γ response to RD1 proteins in prolonged (long-term response) whole blood tests in those subjects resulting negative to assays such as QuantiFERON-TB Gold In tube (QFT-IT).

**Methods:**

The study population included 106 healthy TST^+ ^individuals with suspected LTBI (recent contact of smear-positive TB and homeless) consecutively enrolled. As controls, 13 healthy subjects unexposed to *M. tuberculosis *(TST^-^, QFT-IT^-^) and 29 subjects with cured pulmonary TB were enrolled. IFN-γ whole blood response to RD1 proteins and QFT-IT were evaluated at day 1 post-culture. A prolonged test evaluating long-term IFN-γ response (7-day) to RD1 proteins in diluted whole blood was performed.

**Results:**

Among the enrolled TST^+ ^subjects with suspected LTBI, 70/106 (66.0%) responded to QFT-IT and 64/106 (60.3%) to RD1 proteins at day 1. To evaluate whether a prolonged test could improve the detection of LTBI, we set up the test using cured TB patients (with a microbiologically diagnosed past pulmonary disease) who resulted QFT-IT-negative and healthy controls as comparator groups. Using this assay, a statistically significant difference was found between IFN-γ levels in cured TB patients compared to healthy controls (p < 0.006). Based on these data, we constructed a receiver operating characteristic (ROC) curve and we calculated a cut-off. Based on the cut-off value, we found that among the 36 enrolled TST+ subjects with suspected LTBI not responding to QFT-IT, a long term response to RD1 proteins was detected in 11 subjects (30.6%).

**Conclusion:**

These results indicate that IFN-γ long-term response to *M. tuberculosis *RD1 antigens may be used to detect past infection with *M. tuberculosis *and may help to identify additional individuals with LTBI who resulted negative in the short-term tests. These data may provide useful information for improving immunodiagnostic tests for tuberculosis infection, especially in individuals at high risk for active TB.

## Background

In recent years, several immunodiagnostic assays have been developed for diagnosing *Mycobacterium tuberculosis *(*M. tuberculosis*) infection [1,2]. These assays, referred as interferon (IFN)-γ release assays (IGRAs), have been specifically designed to overcome the problem of low specificity of the tuberculin skin test (TST). In fact, they detect cellular immune response to antigens which are absent in Bacille Calmètte Guerin (BCG) and most environmental mycobacteria, and specifically present in *M. tuberculosis*. Two such antigens, early-secreted antigenic target (ESAT)-6 and culture filtrate protein (CFP)-10, encoded in the mycobacterial genomic region of difference (RD)-1 were first evaluated in a 6-day lymphocyte stimulation test (LST) and found to be sensitive and specific for diagnosing tuberculosis (TB) [3-7]. Subsequently, other IGRAs were developed that differed from the classical LST with respect to the *in vitro *incubation period, the type of cells cultured [whole blood, frozen or fresh peripheral blood mononuclear cells (PBMC)] and the way that the IFN-γ response is detected [(by enzyme-linked immunosorbent assay (ELISA) or enzyme-linked immunospot assay (ELISPOT)].

A number of clinical studies found discrepancies between TST and IGRAs that were mostly attributed to TST positivity associated with prior BCG vaccination [8-10]. However, data from recent studies [11-13] indicate that this explanation may not account for all discrepant results, as a substantial group of BCG-unvaccinated subjects with TST indurations of >15 mm had negative results by commercially available IGRAs, the QuantiFERON-TB Gold In-tube (QFT-IT) test and/or the T-SPOT. *TB *test (Oxford Immunotec, Abingdon, United Kingdom) [11-13]. These data suggest that IGRAs may have a suboptimal sensitivity, and indicate the need to develop new tools to increase their accuracy.

Prolonged whole blood stimulation has been used to detect memory response in vaccine trials [14]. Using RD1 proteins, it has also been employed to identify subjects with LTBI comparing the results obtained with those found by short-term ELISPOT [15].

The aim of this study was to evaluate if IFN-γ response to RD1 antigens by prolonged (long-term response, 7-day) whole blood stimulation could improve identification of LTBI in subjects resulting negative to QFT-IT. The study population included 106 healthy Human Immunodefciency Vius (HIV)-negative TST^+ ^individuals exposed to *M. tuberculosis *(recent contacts of smear positive pulmonary TB patients) or at higher likelihood to have LTBI, such as the homeless population, as they are at higher risk to develop active TB disease compared to the general population [16]. Controls included 13 TST^- ^QFT-IT^-^unexposed individuals and 29 cured TB subjects. All the subjects were consecutively enrolled.

## Methods

### Study population

At the "L. Spallanzani" National Institute for Infectious Diseases (INMI) we consecutively enrolled in this study TST^+ ^individuals with risk factors for TB infection including close contacts in the last 3 months of patients with a positive sputum culture for *M. tuberculosis *and a group of TST^+ ^homeless people known to have been exposed to *M. tuberculosis *in the past. Chemoprophylaxis was offered to all subjects, thereafter referred to as suspected LTBI, but none of them was under chemoprophylaxis at the time of the study. As control groups, we enrolled: 1) "controls", individuals with no risk of *M. tuberculosis *infection who tested TST-negative and QFT-IT-negative; 2) "cured tuberculosis", individuals with culture-positive pulmonary tuberculosis at the time of diagnosis and who were culture-negative upon treatment completion when the draw blood was performed.

Individuals who tested positive to HIV-antibody assay or who were on immunosuppressive drugs were not included in the study. Upon enrolment, demographic and epidemiological information was collected by a physician through a structured questionnaire, including information about BCG vaccination.

The study was approved by the INMI ethical committee, and all enrolled individuals provided written consent.

### TST

Participants underwent a TST on the day of blood sampling (see below). The TST was administered by the Mantoux procedure using 5 IU of tuberculin (Chiron, Siena, Italy). Results were read after 72 hrs. Induration of at least 5 mm was considered a positive response for the group of close contacts and a reaction of ≥ 10 mm was considered positive for homeless people and controls [17].

#### Commercially available IGRA

QuantiFERON TB-Gold In tube (Cellestis Limited) was used. The tube containing TB antigen uses overlapping peptides from CFP-10 and ESAT-6 [18] and TB7.7. The assay was performed and the results were scored as indicated by the manufacturer (cut-off value for a positive test was 0.35 IU/ml). For IFN-γ values above 10 IU/ml serial dilutions of plasma were performed.

### Whole blood cultures with *M. tuberculosis *antigens and mitogen

#### 1-day (short-term) response

0.5 ml per well of heparinized whole blood was seeded in a 48-well plate (Corning Costar, Corning Incorporated, New York, NY, USA) and treated with RD1 intact proteins (ESAT-6 and CFP-10) at 0.2 μg/ml each protein (Lionex, Braunschweig, Germany) and the mitogen phytohaemagglutinin (PHA) at 5 μg/ml (Sigma, St Louis, MO, USA). Samples were incubated for 24 hrs. At day 1, plasma was harvested and cold stored until tested (+4°C or if used after 15 days at -20°C).

#### 7-day (long-term) response

We used the described methodology [14,15]. Briefly at the day of sampling, an aliquot of blood was diluted 5-fold using RPMI 1640 supplemented with penicillin, streptomycin and 2 mM L-glutamine (all four products are from Euroclone Ltd, United Kingdom) and was plated into 48-well plates (Corning) and stimulated as described above. The day-7 diluted plasma was harvested following incubation at 37°C and cold stored until tested (+4°C or if used after 15 days at -20°C).

#### IFN-γ determination

IFN-γ from day-1 and day-7 plasma were evaluated by a commercial ELISA (CMI, Cellestis Limited, Carnegie, Victoria, Australia) and are presented as IU/ml after subtraction of the appropriate control. For day-1 analysis, a cut-off value was previously determined by constructing a Receiver Operator Characteristic (ROC) curve by means of LABROC-1 software and was 0.7 IU/mL for all stimuli [19,20]

### Statistical analysis

The main outcome of the study was the evaluation of IFN-γ production in response to the mitogen PHA and antigenic stimulation in the QFT-IT and whole blood assays (WBA), expressed as dichotomous (positive/negative) and continuous (IU/ml) measures. Median and interquirtile range (IQR) of IFN-γ production were calculated. The Mann-Whitney U test was used to compare continuous variables and Chi square or McNemar tests were used for categorical variables. The cut-off value for definition of positivity of the WBA based on RD1 proteins at day 7 was defined by ROC analysis. SPSS v 14 for Windows (SPSS Italia srl, Bologna, Italy) and Prism 4 software (Graphpad Software 4.0, San Diego, CA, USA) were used in the analysis.

## Results

### Characteristics of the population

We studied a population of TST^+ ^subjects with risk factors for LTBI, 54 healthy contacts of patients with a positive pulmonary TB smear and 52 homeless people. No differences were found in terms of the characteristics reported and therefore the data were pooled together. The population is referred to as suspected LTBI. As controls, we used cured TB subjects and healthy individuals unexposed to *M. tuberculosis *who resulted negative to TST and QFT-IT. The characteristics of the groups are reported in table 1. Note that among the suspected LTBI subjects, the TST median was high (20 mm).

**Table 1:** Characteristics of study subjects.

	Controls N (%) 13 (8.8)	**TST**^+^, **suspected LTBI** N (%) 106 (71.6)	Cured TB N (%) 29 (19.6)	Total N (%) 148 (100)
**Age Median (IQR)**	33 (29.5-35)	35 (21-48)	31.5 (25-49)	36 (28-50)
**Female Gender**	8 (61.5)	33 (31.1)	15 (51.7)	56 (37.8)
				
**Origin**				
**Italy**	12 (92.3)	49 (46.3)	9 (31.1)	70 (47.3)
**Abroad**	1 (7.7)	57 (53.7)	20 (68.9)	78 (52.7)
**TST Median in mm (IQR)**	0 (0)	20 (15-45)	ND	20* (15-30)
**TST**^+^	0	106 (100)	ND	106/119** (89)
**BCG**				
**Vaccinated**	4 (30.8)	37 (34.9)	19 (65.5)	60 (40.6)
**unvaccinated** **unknown**	9 (69.2) 0	50 (47.2) 19 (17.9	10 (34.5) 0	69 (46.6) 19 (12.8)

**Footnotes**: BCG: Bacillus Calmètte et Guerin; LTBI: latent tuberculosis infection; N: number; TST: tuberculin skin test.

*among TST+

** among those tested by TST

### Responses to QFT-IT and RD1 proteins by short-term stimulation

All the enrolled subjects responded to PHA (data not shown). As for the selection performed, none of the controls responded to the QFT-IT and to RD1 proteins. The median of IFN-γ production in response to QFT-IT was 0 IU/mL (IQR: 0-0) and to RD1 proteins 0 IU/mL (IQR: 0-0.1).

Among the enrolled TST^+ ^subjects with suspected LTBI, the response to QFT-IT was found in 66.0% (70/106), whereas to RD1 proteins in 60.3% (64/106) (table 2). Among all of the subjects of this group, the median of IFN-γ production in response to QFT-IT was 2.3 IU/mL (IQR: 0-15.1) and to RD1 proteins 1.4 IU/mL (IQR: 0.2-8.9). Among the responders, the median of IFN-γ production in response to QFT-IT was 8.1 IU/mL (IQR: 2.4-22.6) and to RD1 proteins 7.6 IU/mL (IQR: 2.3-20.3).

**Table 2:** *In vitro *responses to *M. tuberculosis *antigens in the study group populations.

*In vitro* **response to**:		Controls N (%)	**TST**^+^, **suspected LTBI** N (%)	Cured TB N (%)
		13 (100)	106 (100)	29 (100)
**QFT-IT**				
	**Positive N.(%)**	0	70 (66.03)	19 (63.3)
	**IFN-γ (IU/ml) median (IQR)** **Among all**	0 (0-0.1)	2.3 (0-15.1)	1.1 (0.1-4.2)
	**IFN-γ (IU/ml) median (IQR)** **Among the positive responders**	NA	8.1 (2.4-22.6)	3.8 (1.5-5.0)
				
**RD1 proteins**				
	**Positive N.(%)**	0	64 (60.3)	19 (65.5)
	**IFN-γ (IU/ml) median (IQR)** **Among all**	0 (0-0.2)	1.4 (0.2-8.9)	1.6 (0.4-2.9)
	**IFN-γ (IU/ml) median (IQR)** **Among the positive responders**	NA	7.6 (2.3-20.3)	2.6 (1.6-6.2)

Footnotes: IQR: inter quartile range; LTBI: latent tuberculosis infection; N: number; QFT-IT: QuantiFERON-TB Gold In-tube; RD: region of difference, TB: tuberculosis.

Among the cured TB subjects, the response to QFT-IT and RD1 proteins was found for both tests in 65.5% (19/29). The median of IFN-γ production in response to QFT-IT was 1.1 IU/mL (IQR: 0.1-4.2) and to RD1 proteins 1.6 IU/mL (IQR: 0.4-2.9). Among the responders, the median of IFN-γ production in response to QFT-IT was 3.8 IU/mL (IQR: 1.5-5.0) and to RD1 proteins 2.6 IU/mL (IQR: 1.6-6.2).

No statistical difference was found between the enrolled subjects with suspected LTBI and cured TB in response to QFT-IT and RD1 proteins, in terms of both qualitative and quantitative response (p > 0.05). However, a significant statistical difference was found between controls and both groups of suspected LTBI and cured TB in response to QFT-IT and RD1 proteins, in terms of both qualitative and quantitative response (p < 0.0001 for all comparisons).

### Long-term response to RD1 proteins

In an effort to find new tools for detecting RD1-specific response in those resulting negative to QFT-IT and to RD1 proteins at day-1 post stimulation, we evaluated long-term-specific IFN-γ production. We used the RD1 proteins as antigenic stimulus because previously used for prolonged WBA [15], because we could plate the diluted blood and also because the results obtained by RD1 proteins stimulation were similar to those obtained by QFT-IT test, as reported above (Table 2) and in previous studies [19,20].

**Figure 1:**
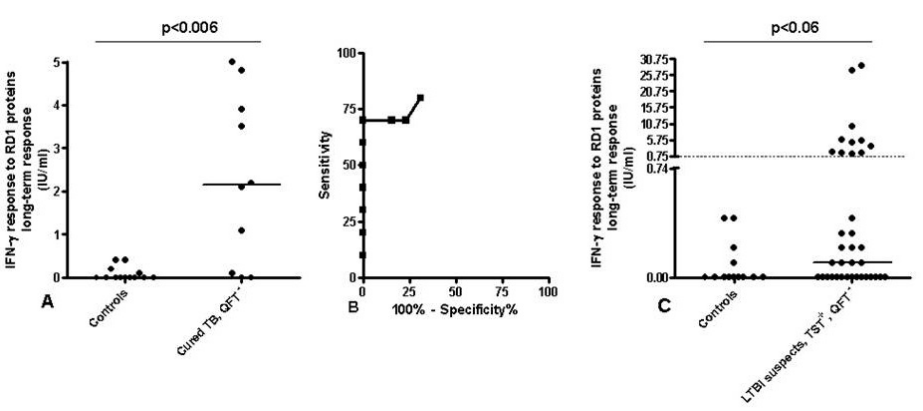
**A-B-C. Long-term response to RD1 proteins in cured TB subjects and suspect of LTBI resulted negative to QFT-IT compared to healthy controls**. IFN-γ production in response to RD1 proteins was evaluated at day 7 in diluted whole blood of subjects who resulted QFT-IT-negative as described in the material and method section. **A) **IFN-γ release was significantly higher in cured TB patients than healthy controls (p < 0.006). Horizontal lines indicate the median IFN-γ production. The data are presented as IU/mL. **B)**. A ROC analysis was performed using the established cured TB group and the healthy controls group as comparator groups. **C) **IFN-γ production in response to RD1 proteins was higher, although not significantly, compared to controls (p < 0.06). Eleven out of thirty-six (30.6%) responded to RD1 proteins. Horizontal lines indicate the median IFN-γ production. The dotted line indicates the cut-off. The data are presented as IU/mL.

As reported in the literature, IGRA scoring may become negative after successful tuberculosis therapy in diseased patients [21-23]. Therefore, we evaluated whether long-term stimulation could be a good approach for detecting a memory response in cured TB subjects at the time of therapy completion that resulted negative to QFT-IT. Within the 29 subjects with cured TB enrolled in the study, 10 (34.4%) resulted QFT-IT-negative. In this subgroup of subjects, the long-term IFN-γ response to RD1 proteins was significantly higher (p < 0.006; median: 2.1; IQR: 0.050-4.3) compared to healthy controls (median: 0; IQR: 0-0.15) (Figure 1A). Based on these data, we performed a ROC analysis using the QFT-IT-negative cured TB subjects and the healthy controls as comparator groups (Figure 1B). Significant results for area under the curve (AUC) analysis were obtained [AUC, 0.84; confidence interval (CI), 0.65-1.02, p < 0.005]. For scoring purposes we chose a cut-off to maximize the sum of sensitivity and specificity. We found that a cut-off of 0.75 (IU/ml) predicted a long term response in cured TB with 70.0% sensitivity (95% CI, 34.75%-93.33%) and 100% specificity (95% CI, 75.29%-100.0%).

Based on this cut-off value we scored the results as negative and positive. None of the controls resulted positive, whereas among the cured TB subjects not responding to QFT-IT, 7/10 (70%) scored positive (Figure 1A). Among those with suspected LTBI who did not respond to QFT-IT 11/36 (30.6%) responded to the long-term test (Figure 1C). Moreover, in this subgroup, a higher, although not significant level of IFN-γ production (p < 0.06) was found in response to RD1 proteins (median: 0.1, IQR 0-1.65) compared to controls.

We then evaluated the impact of BCG vaccination in the response to the prolonged test in the suspected LTBI subjects not responding to QFT-IT (Table 3). Among those BCG-unvaccinated 5/14 (35.7%) responded to the prolonged test, among those BCG-vaccinated 3/15 (20.0%) responded and among those with BCG status unknown 3/7 (42.9%) responded. Based on these data, no clear association was found between BCG vaccination and lack of response to the long-term test.

**Table 3:** Impact of BCG vaccination on the results obtained by the prolonged test.

	TST^+^, suspected LTBI, not responding to QFT-IT			
	**BCG**			
**Response to the prolonged test**	**Unvaccinated** **N. 14 (%)**	**Vaccinated** **N. 15 (%)**	**Unknown** **N.7 (%)**	**Total** **N. 36 (%)**

**No responders N. 25**	9 (64.3)	12 (80.0)	4 (57.1)	25 (69.4)
**Responders N. 11**	5 (35.7)	3 (20.0)	3 (42.9)	11 (30.6)

Footnotes: BCG: Calmette et Guerin; LTBI: latent tuberculosis infection; N: number; QFT-IT: QuantiFERON TB-Gold-In tube; TST: tuberculin skin test.

## Discussion

In this study we demonstrated that IFN-γ long-term response to *M. tuberculosis *RD1 proteins may be detected in TST^+ ^individuals who, although with a high likelihood of having LTBI, resulted negative to a commercial IGRA. Among these individuals, we found a specific-IFN-γ long-term response in 11/36 (30.6%). A higher proportion of positive responses (7/10, 70.0%) was found among patients with cured TB, while no positive responses were detected among low risk controls. These observations suggest that an IFN-γ long-term response may indeed be associated with TB infection. These results provide useful information on the immunity against *M. tuberculosis *and may be the preliminary basis for further improvement of immunodiagnostic tests for LTBI. In particular it may be hypothesized that a long-term test can be used in a series with a "conventional IGRA" when a high sensitivity for LTBI detection is required, such as in the screening of candidates for an immunosuppressive treatment.

In absence of a gold standard for diagnosing LTBI, identifying a population of individuals with this condition is not straightforward. However, we believe that the TST^+ ^individuals enrolled in the present study were very likely to have been infected with *M. tuberculosis *for two reasons: first, they all had high risk factors for TB infection and second, they had a great TST response which is most likely associated to a past infection, even among those who are BCG-vaccinated [24]. Thus, the observation that almost 34% of the these TST^+ ^individuals had negative results by QFT-IT may reflect, at least in part, a less than perfect sensitivity to this test for detecting LTBI. This observation is consistent with previous reports [10,15,25,26]. In particular, in two cross-sectional studies performed in South Africa, approximately one-third of adults with a TST induration higher than 15 mm had a negative QFT-IT test result and 38% had a negative T-SPOT.TB test result [25,27]. Another study reported that in a mostly BCG-vaccinated Korean control population, 51% of the subjects were TST^+ ^and only 4% were QFT-IT positive, while the expected prevalence of *M*. *tuberculosis *infection was 33% [26].

We performed short and prolonged tests using identical *M. tuberculosis-*specific proteins (RD1) that were found to be highly specific for *M*. *tuberculosis *[1,2,11,25] to provide consistent results. By this approach, one third of the TST^+ ^individuals who had negative results by QFT-IT or RD1 proteins-based short-term assay resulted positive. Among the participants who scored negative on short test, high levels of IFN-γ could be detected by the prolonged test, indicating that the observed discrepancy was not simply explained by differences in the levels of detection of IFN-γ. Another plausible explanation for the difference in sensitivity would be the diversities in the *in vitro *incubation periods for the QFT-IT and RD1 proteins-based short test and prolonged assay. Probably the long term incubation leads to higher likelihood of IFN-γ production since the protein processing may be performed by a higher number of T cell populations with various T-cell receptors types with consequently a better antigen presentation than in short term culture (1-day). Moreover, we hypothesize that after 24 hours of incubation, only circulating effector memory T-cells had sufficient time to produce IFN-γ, while central memory T-cells or T cells in resting state first started producing IFN-γ after a more prolonged incubation needed to allow them to differentiate. In individuals who had been infected with *M. tuberculosis *in the past, the number of circulating effector cells could be low, causing negative results in a short-incubation assay but positive responses after a prolonged incubation, as reported by our studies and by others [11,28].

In accordance with this line of thought, findings from a hepatitis C virus study show that short-term ELISPOT responses were not influenced by depletion of lymphotropic chemokine receptor 7-positive T-cells, representing memory cells, while the depletion of these memory cells did decrease the antigen-specific responses after prolonged culture [29].

There is a lack of knowledge of the performance of IGRAs with a short-term incubation period compared to those with a more prolonged incubation time for detecting *M. tuberculosis *infection. To our knowledge, there are few studies comparing the overnight ELISPOT or WBA with either the 6-day LST or the 7-day whole blood incubation test and the results indicate that the prolonged tests performed better [11,15,30]. This is also in agreement with our previous data in which we reported that negative responses to a panel of RD1 peptides in an overnight ELISPOT became positive responses in a cultured ELISPOT [28].

In this study, 69.4% (25/36) of the enrolled TST+ subjects with suspected LTBI who did not respond to QFT-IT, did not respond to the long-term assay either. This was also reported in the cured TB subjects enrolled, although in a lower proportion (3/10, 30%). Further research is needed to elucidate this lack of specific response.

This is a pilot study which presents limitations that will require additional work to be overcome. Above all, the study was performed on a relatively small number of individuals and certainly the research would benefit from a larger study. Other limitations include the self-reported BCG vaccination which may account for potential mistakes and imprecise information, the potential exposure to environmental mycobacteria which may have caused a cross-reaction with the tuberculin preparation present in the TST and finally the amount of the refusal rate among the homeless [refusal rate was 4.3% (13/300) at recruitment, 10.1% (29/287) for the TST reading, and 11.8% (7/59) to undergo further investigation for those resulted TST+] that may have accounted for a selection of the subjects tested.

## Conclusion

In conclusion, IFN-γ long-term response to *M. tuberculosis *specific antigens among TST^+ ^individuals who resulted negative to QFT-IT can potentially be considered to be an additional tool for detecting LTBI. Further studies are needed to confirm these data in a larger number of patients and to evaluate the possible implications in terms of TB risk, on the positivity of short vs. prolonged tests.

## Abbreviations

AUC: area under the curve; BCG: Bacillus Calmette-Guérin; CFP-10: culture filtrate protein-10 kDa; CI: confidence interval; ELISA: Enzyme Linked ImmunoSorbent Assay; ELISPOT: enzyme-linked immunospot assay; ESAT-6: early secretory antigenic target-6; HIV: human immunodeficiency virus; IFN: interferon; IGRAs: Interferon gamma release assay; INMI: National Institute for Infectious Diseases; IQR: interquartile range; LST: lymphocyte stimulation test; LTBI: latent tuberculosis infection; *M. tuberculosis: Mycobacterium tuberculosis; *PBMC: peripheral blood mononuclear cells; PHA: phytohaemagglutinin; QFT-IT: QuantiFERON TB-Gold In tube; RD1: region of difference 1; ROC: Receiver operating characteristic; TB: Tuberculosis; TST: Tuberculin skin test; WBA: Whole blood assays.

## Competing interests

The authors declare that they have no competing interests.

## Authors' contributions

OB carried out the experimental data and analysis, drafted and revised the manuscript; TC, and VV carried out the Quantiferon -TB-Gold In tube assay; SM, GGNC, FD, SC, RC were responsible for the experimental data analysis; MS provided RD1 reagents; EG and DG were responsible for the statistical analysis; MV, FNL, AM, PL, DG were responsible for patient selection and enrolment; DG and EG were responsible for the conception of the study design and the preparation of the manuscript. All authors read and approved the final manuscript.

## Pre-publication history

The pre-publication history for this paper can be accessed here:

http://www.biomedcentral.com/1471-2334/9/182/prepub

## References

[B1] Pai M Zwerling A Menzies D Systematic review: T-cell-based assays for the diagnosis of latent tuberculosis infection: An update Ann Intern Med 2008 149 177 84 18593687 10.7326/0003-4819-149-3-200808050-00241 PMC2951987

[B2] Goletti D Carrara S Butera O Amicosante M Ernst M Sauzullo I Vullo V Cirillo D Borroni E Markova R Drenska R Dominguez J Latorre I Angeletti C Navarra A Petrosillo N Lauria FN Ippolito G Migliori GB Lange C Girardi E Accuracy of immunodiagnostic tests for active tuberculosis using single and combined results: a multicenter TBNET-Study PLoS ONE 2008 3 e3417 10.1371/journal.pone.0003417 18923709 PMC2561073

[B3] Arend SM Andersen P van Meijgaarden KE Skjot RL Subronto YW van Dissel JT Ottenhoff TH Detection of active tuberculosis infection by T cell responses to early-secreted antigenic target 6-kDa protein and culture filtrate protein 10 J Infect Dis 2000 181 1850 1854 10.1086/315448 10823800

[B4] Munk ME Arend SM Brock I Ottenhoff TH Andersen P Use of ESAT-6 and CFP-10 antigens for diagnosis of extrapulmonary tuberculosis J Infect Dis 2001 183 175 176 10.1086/317663 11106545

[B5] Ravn P Demissie A Eguale T Wondwosson H Lein D Amoudy HA Mustafa AS Jensen AK Holm A Rosenkrands I Oftung F Olobo J von Reyn F Andersen P Human T cell responses to the ESAT-6 antigen from *Mycobacterium tuberculosis* J Infect Dis 1999 179 637 645 10.1086/314640 9952370

[B6] Wu-Hsieh BA Chen CK Chang JH Lai SY Wu CH Cheng WC Andersen P Doherty TM Long-lived immune response to early secretory antigenic target 6 in individuals who had recovered from tuberculosis Clin Infect Dis 2001 33 1336 1340 10.1086/323044 11565073

[B7] Mahairas GG Sabo PJ Hickey MJ Singh DC Stover CK Molecular analysis of genetic differences between Mycobacterium bovis BCG and virulent *M. bovis* J Bacteriol 1996 178 1274 82 8631702 10.1128/jb.178.5.1274-1282.1996 PMC177799

[B8] Ewer K Deeks J Alvarez L Bryant G Waller S Andersen P Monk P Lalvani A Comparison of T-cell-based assay with tuberculin skin test for diagnosis of *Mycobacterium tuberculosis *infection in a school tuberculosis outbreak Lancet 2003 361 1168 1173 10.1016/S0140-6736(03)12950-9 12686038

[B9] Lalvani A Pathan AA Durkan H Wilkinson KA Whelan A Deeks JJ Reece WH Latif M Pasvol G Hill AV Enhanced contact tracing and spatial tracking of *Mycobacterium tuberculosis *infection by enumeration of antigen-specific T cells Lancet 2001 357 2017 2021 10.1016/S0140-6736(00)05115-1 11438135

[B10] Shams H Weis SE Klucar P Lalvani A Moonan PK Pogoda JM Ewer K Barnes PF Enzyme-linked immunospot and tuberculin skin testing to detect latent tuberculosis infection Am J Respir Crit Care Med 2005 172 1161 1168 10.1164/rccm.200505-748OC 16081545 PMC2718400

[B11] Leyten EM Arend SM Prins C Cobelens FG Ottenhoff TH van Dissel JT Discrepancy between Mycobacterium tuberculosis-specific gamma interferon release assays using short and prolonged *in vitro *incubation Clin Vaccine Immunol 2007 14 880 5 10.1128/CVI.00132-07 17507543 PMC1951056

[B12] Arend SM Thijsen SF Leyten EM Bouwman JJ Franken WP Koster BF Cobelens FG van Houte AJ Bossink AW Comparison of two interferon-gamma assays and tuberculin skin test for tracing TB contacts Am J Respir Crit Care Med 2007 175 618 627 10.1164/rccm.200608-1099OC 17170386

[B13] Leyten EM Prins C Bossink AW Thijsen S Ottenhoff TH van Dissel JT Arend SM Effect of tuberculin skin testing on a *Mycobacterium tuberculosis*-specific IFN-γ assay Eur Respir J 2007 29 1212 6 10.1183/09031936.00117506 17215314

[B14] Black GF Dockrell HM Crampin AC Floyd S Weir RE Bliss L Sichali L Mwaungulu L Kanyongoloka H Ngwira B Warndorff DK Fine PE Patterns and implications of naturally acquired immune responses to environmental and tuberculous mycobacterial antigens in northern Malawi J Infect Dis 2001 1 184 322 9 10.1086/322042 11443558

[B15] American Thoracic Society Diagnostic standards and classification of tuberculosis in adults and children Am J Respir Crit Care Med 2000 161 1376 95 10764337 10.1164/ajrccm.161.4.16141

[B16] McAdam JM Bucher SJ Brickner PW Vincent RL Lascher S Latent tuberculosis and active tuberculosis disease rates among the homeless Emerg Infect Dis 2009 15 1109 11 10.3201/eid1507.080410 19624932 PMC2744228

[B17] Lawn SD Bangani N Vogt M Bekker LG Badri M Ntobongwana M Dockrell HM Wilkinson RJ Wood R Utility of interferon-gamma ELISPOT assay responses in highly tuberculosis-exposed patients with advanced HIV infection in South Africa BMC Infect Dis 2007 7 99 10.1186/1471-2334-7-99 17725839 PMC2031899

[B18] Mori T Sakatani M Yamagishi F Takashima T Kawabe Y Nagao K Shigeto E Harada N Mitarai S Okada M Suzuki K Inoue Y Tsuyuguchi K Sasaki Y Mazurek GH Tsuyuguchi I Specific detection of tuberculosis infection: an interferon-gamma-based assay using new antigens Am J Respir Crit Care Med 2004 170 59 64 10.1164/rccm.200402-179OC 15059788

[B19] Goletti D Parracino MP Butera O Bizzoni F Casetti R Dainotto D Anzidei G Nisii C Ippolito G Poccia F Girardi E I.soniazid prophylaxis differently modulates T-cell responses to RD1-epitopes in contacts recently exposed to Mycobacterium tuberculosis a pilot study Respir Res 2007 8 5 10.1186/1465-9921-8-5 17257436 PMC1794408

[B20] Goletti D Carrara S Vincenti D Saltini C Rizzi EB Schininà V Ippolito G Amicosante M Girardi E Accuracy of an immune diagnostic assay based on RD1 selected epitopes for active tuberculosis in a clinical setting: a pilot study Clin Microbiol Infect 2006 12 544 50 10.1111/j.1469-0691.2006.01391.x 16700703

[B21] Aiken AM Hill PC Fox A McAdam KP Jackson-Sillah D Lugos MD Donkor SA Adegbola RA Brookes RH Reversion of the ELISPOT test after treatment in Gambian tuberculosis cases BMC Infect Dis 2006 30 6 66 10.1186/1471-2334-6-66 PMC1562425 16573826

[B22] Carrara S Vincenti D Petrosillo N Amicosante M Girardi E Goletti D Use of a T cell-based assay for monitoring efficacy of antituberculosis therapy Clin Infect Dis 2004 38 754 6 10.1086/381754 14986262

[B23] Sauzullo I Mengoni F Lichtner M Massetti AP Rossi R Iannetta M Marocco R Del Borgo C Soscia F Vullo V Mastroianni CM In vivo and in vitro effects of antituberculosis treatment on mycobacterial Interferon-gamma T cell response PLoS One 2009 4 e5187 10.1371/journal.pone.0005187 19365543 PMC2664463

[B24] Farhat M Greenaway C Pai M Menzies D False-positive tuberculin skin tests: what is the absolute effect of BCG and non-tuberculous mycobacteria? Int J Tuberc Lung Dis 2006 10 1192 204 17131776

[B25] Rangaka MX Wilkinson KA Seldon R Van Cutsem G Meintjes GA Morroni C Mouton P Diwakar L Connell TG Maartens G Wilkinson RJ The effect of HIV-1 infection on T cell based and skin test detection of tuberculosis infection Am J Respir Crit Care Med 2007 175 514 520 10.1164/rccm.200610-1439OC 17158278

[B26] Kang YA Lee HW Yoon HI Cho B Han SK Shim YS Yim JJ Discrepancy between the tuberculin skin test and the whole-blood interferon gamma assay for the diagnosis of latent tuberculosis infection in an intermediate tuberculosis-burden country JAMA 2005 293 2756 2761 10.1001/jama.293.22.2756 15941805

[B27] Mahomed H Hughes EJ Hawkridge T Minnies D Simon E Little F Hanekom WA Geiter L Hussey GD Comparison of Mantoux skin test with three generations of a whole blood IFN-gamma assay for tuberculosis infection Int J Tuberc Lung Dis 2006 10 310 316 16562712

[B28] Goletti D Butera O Bizzoni F Casetti R Girardi E Poccia F Region of difference 1 antigen-specific CD4_ memory T cells correlate with a favorable outcome of tuberculosis J Infect Dis 2006 194 984 992 10.1086/507427 16960787

[B29] Godkin AJ Thomas HC Openshaw PJ Evolution of epitope-specific memory CD4_ T cells after clearance of hepatitis C virus J Immunol 2002 169 2210 2214 12165552 10.4049/jimmunol.169.4.2210

[B30] Moskophidis D Lechner F Pircher H Zinkernagel RM Virus persistence in acutely infected immunocompetent mice by exhaustion of antiviral cytotoxic effector T cells Nature 1993 362 758 61 10.1038/362758a0 8469287

